# Which one to choose? A cost-effectiveness analysis between different technologies of air purifiers

**DOI:** 10.1093/eurpub/ckac131.250

**Published:** 2022-10-25

**Authors:** C Gullotto, M Sparacino, A Amodeo, G Cevenini, N Nante, G Messina

**Affiliations:** 1 Hygiene and Preventive Medicine, University of Siena, Siena, Italy; 2 Department of Medical Biotechnologies, University of Siena, Siena, Italy; 3 Department of Molecular and Developmental Medicine, University of Siena, Siena, Italy

## Abstract

**Introduction:**

CoViD19 pandemic highlighted the importance of air purifiers and, in commercialization, their performance and price influence the choice. Since primary focus concerns only performance in terms of CADR (Clean Air Delivery Rate), this study aims to compare: I) levels of declared air purifications according to different types of air purification technologies; II) price of them to evaluate if, with similar group-mean CADR (within +/- 1 SD), there are significant differences in selling prices.

**Methods:**

A review of several devices was carried out, collecting data in January-April 2022. Four different types of air purifiers were considered, divided into as many groups: those equipped with HEPA filters + UV lamps, only with HEPA filters, only with UV lamps and those using other technologies. We applied Kruskal-Wallis test to evaluate statistical differences among prices normalized by CADR, at significant level of 0.05.

**Results:**

Analysis was carried out on 186 devices: I) 37 had HEPA filters + UV lamps, II) 117 only HEPA filters, III) 11 only UV lamps and IV) 21 other technologies. Eight system had HEPA H11 (95% reduction of particle matter 0.5 μm), 8 had HEPA H12 (99.5%), 70 had HEPA H13 (99.95%), 11 had HEPA H14 (99.995%). The mean normalized costs of each group devices, in Euros/CADR were I) 1.22 (SD 2), II) 1.49 (SD 1.4), III) 7.63 (SD 7.38), IV) 1.22 (SD 0.99), respectively. Statistical comparison of four-group selling prices show significant differences (p < 0.05) due to the devices equipped with only UV lamps.

**Conclusions:**

Comparison between technologies analyzed by mean price normalized to CADR showed significant differences between those that used only UV lamps compared to all the others. This is reasonably due to the fact that the use of only UV lamps requires radiant powers considerably greater than all the others, therefore also higher costs (about 5-6 times). In all cases, the level of disinfection reached, as declared, was always > 95%.

**Key messages:**

• With the same mean price normalized to CADR, the selling price is significanly different only for devices equipped with UV lamps compared to all the others.

• Choice of devices with a certain level of declared air purifications can be directed towards those with HEPA+UV/HEPA/other without the mean price normalized to CADR undergoing significant differences.

